# Study of fatigue damage behavior in off-axis CFRP composites using digital image correlation technology

**DOI:** 10.1016/j.heliyon.2024.e25577

**Published:** 2024-02-05

**Authors:** Zhendong Zhong, Fusheng Wang, Fanqi Kong, Yajun Chen

**Affiliations:** Sino-European Institute of Aviation Engineering, Civil Aviation University of China, Tianjin, 300300, China

**Keywords:** Off-axis CFRP composites, Digital image correlation, Fatigue, Damage behavior

## Abstract

This study investigates the fatigue behavior of off-axis carbon fiber reinforced polymer (CFRP) composites under varying stress levels, with a focus on both tensile-tensile and compressive-compressive loading modes. We conduct a comprehensive analysis of energy dissipation and stiffness across various loading conditions, highlighting the critical role of fiber deflection effects in the recovery of tensile-tensile fatigue properties. Utilizing digital image correlation (DIC) technology, we identify both commonalities and distinctions in crack propagation pathways and failure mechanisms between these two fatigue scenarios. In the case of tensile-tensile fatigue, the predominant damage mechanism involves the development of multiple interlaminar shear cracks. These cracks initiate debonding at the fiber/resin interface, propagating from macrocracks at the edges to microcracks at the center, ultimately culminating in fiber pull-out failure. Conversely, in compressive fatigue, damage occurs centrally in the form of intralaminar shear cracks. As damage accumulates, these cracks progressively propagate along the fibers towards the edges, accompanied by localized fiber buckling, ultimately resulting in compressive failure. Furthermore, we determine a critical compressive strain threshold, which serves as a pivotal indicator of failure in compressive-compressive fatigue testing for off-axis CFRP composites.

## Highlights

• Investigation of fiber deflection effects.
• Analysis of crack propagation paths using digital image correlation (DIC).
• Comparative fatigue behavior under different loading modes.
• Determination of compressive strain threshold.

## 1 Introduction

Carbon fiber reinforced polymer (CFRP) composites have found widespread applications in various fields such as aerospace, automotive, and renewable energy, owing to their advantages of high strength, high modulus, low density, and corrosion resistance [[1], [2], [3]]. In the aerospace industry, CFRP composites are commonly employed in critical structural elements such as aircraft frame. These components are exposed to intricate cyclic loading conditions during operational service, resulting in the accumulation of fatigue damage within the material and exerting an influence on the overall structural integrity [[4], [5], [6]]. Uniaxial fatigue loading is commonly categorized into three modes: tensile-tensile fatigue, compressive-compressive fatigue, and tensile-compressive fatigue. These distinct loading modes lead to different types of damage, including matrix cracking, fiber buckling, and interface debonding [[7], [8], [9]]. These damage mechanisms result in a degradation of mechanical properties, a reduction in service life, and the potential for sudden failure [10,11]. Consequently, investigating the fatigue damage mechanisms in composite materials is of crucial significance in enhancing the reliability and safety of composite structures [[12], [13], [14]].

Fatigue inherently entails the gradual initiation and propagation of damage. Throughout this process, various modes of damage become intertwined, and complex interplays between them develop over time, resulting in intricacies within fatigue behavior [12,15,16]. The development of fatigue damages is influenced by a range of factors, including material composition, environmental conditions, and loading conditions. Stress levels have been observed to exert a significant influence on fatigue failure mechanisms [[17], [18], [19]]. Vahid et al. [19] investigated fatigue damage in [±45°]_2S_ glass fiber/epoxy composite laminates under tensile-tensile loading. They observed that at high stress levels, the damage was severe and localized and caused fiber pull-out failure at short lifetimes, whereas low stress levels resulted in a more evenly distributed pattern of damage, which led to a longer fatigue life. This suggests that under low stress conditions, fibers efficiently distribute stress uniformly, while high stress levels lead to direct damage to the matrix and interfaces, possibly even causing localized fiber fracture. Additionally, several advanced monitoring techniques have been employed in the investigation of fatigue damage, including acoustic emission [20,21], thermal imaging [[22], [23], [24]], infrared methods [25], and digital image correlation (DIC) technology [26,27]. Chen et al. [20] examined the damage progression during fatigue testing using acoustic emission technology, revealing three stages of damage under high compressive loading fatigue cycles: initial localized damage expansion, subsequent constant rate expansion, and rapid further expansion leading to failure. Kharrat et al. [21] explored the impact of accumulated damage on the acoustic characteristics of local fracture mechanisms in composite materials subjected to cyclic loading. Through experiments conducted under specific stress levels, they established monitoring methods suitable for various fatigue loading levels. Mirzaei et al. [22] assessed the remaining fatigue life of laminated composite materials using thermal imaging technology. They constructed a temperature-damage evolution model, successfully simulating the temperature distribution and damage state in stress concentration zones at different fatigue load levels. Finis et al. [23] tracked changes in fatigue performance using thermal imaging technology and utilized thermal-elastic signals to evaluate stress/strain redistribution and stiffness degradation, revealing trends consistent with mechanical data. Eder et al. [24] introduced an innovative and accurate thermal imaging analysis method. By calculating alterations in thermal image enthalpy values, they effectively identified three fatigue damage stages, demonstrating excellent agreement with hysteresis dissipation curves obtained through mechanical measurements. Fruehmann et al. [25] employed infrared technology to assess the evolution of fatigue damage in woven composite materials, discovering that thermal stress analysis phase data could identify microcracks in woven composite materials, indicating the initiation and presence of fatigue damage. Yoo et al. [26] utilized high-speed servo-hydraulic testing equipment and DIC technology to characterize the dynamic intra-ply fracture toughness of CFRP composite materials. They proposed alternative testing methods for composite materials under medium to high strain rates. Eliasson et al. [27] applied DIC technology to evaluate the fatigue damage process of CFRP materials, finding that the fatigue critical load of Unidirectional CFRP materials is at least 80 % of the ultimate tensile strength (UTS).

Currently, research on fatigue in CFRP composites primarily concentrates on stress-strain responses and stiffness degradation under single, specific fatigue loading modes. However, there is a noticeable deficiency in studies that comprehensively compare fatigue damage behaviors under various loading modes and stress levels. Research on fatigue damage behaviors in materials is also limited, particularly with respect to understanding damage propagation pathways and failure mechanisms. This study investigates the impact of different loading modes and stress levels on energy dissipation and stiffness in composite materials. It highlights the pivotal role of fiber deviation effects in the evolution of fatigue performance under tensile-tensile and compressive-compressive loading. Additionally, by employing DIC technology, we compare fatigue crack propagation paths and failure mechanisms between tensile-tensile and compressive-compressive fatigue. These discoveries not only enhance our comprehension of fatigue failure mechanisms in [45°/-45°]_2s_ off-axis CFRP composites but also provide a theoretical foundation for the design and optimization of off-axis ply configurations in composites.

## 2 Materials and methods

### 2.1 Materials and specimens

**Fig. 1:**
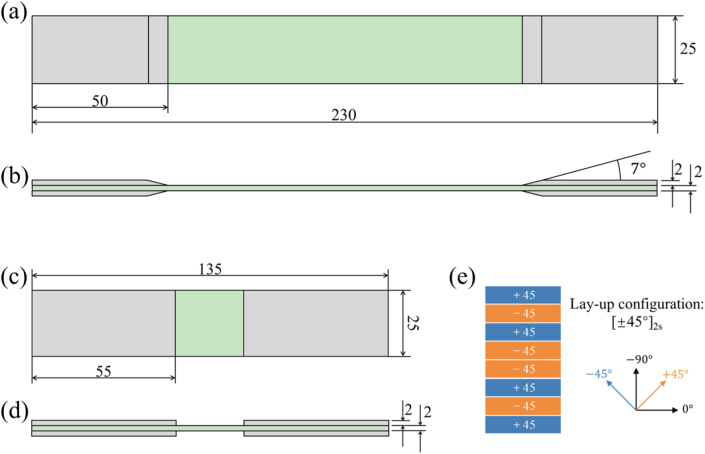
CFRP laminate geometric dimensions, with (a)(b) for tensile-tensile fatigue specimens, (c)(d) for compressive-compressive fatigue specimens, and (e) representing laminate layup.

To emphasize the role of off-axis ply orientations, T700 carbon fiber/epoxy resin specimens with a ply orientation of [45°/-45°]_2s_ were selected. The prepreg, provided by Jiangshan Fiber Technology Co., Ltd., features a resin content of 33 % and an areal weight of 200 g/m^2^. Table 1 lists the essential parameters of the prepreg material. These specimens were fabricated through a hot pressing process, resulting in a cured laminate thickness of 2 mm. The laminates were precisely cut into two types of specimens using a high-pressure waterjet: 230 mm × 25 mm for tensile testing, as shown in Fig. 1(a) and (b), and 135 mm × 25 mm for compression testing, as shown in Fig. 1(c) and (d). The laminate layup as shown in Fig. 1(e). To prevent specimen wear and sliding within the fixtures, aluminum alloy reinforcement sheets, measuring 50 mm × 25 mm and 55 mm × 25 mm, respectively, were securely affixed to both ends of each specimen using polyurethane adhesive.

**Table 1:** Mechanical properties of carbon fiber reinforced epoxy resin prepreg.

E_1_ (MPa)	E_2_ (MPa)	$\nu_{12}$	G_12_ (MPa)	G_13_ (MPa)	G_23_ (MPa)
170000	8100	0.355	4200	4200	3500

### 2.2 Experimental method

#### 2.2.1 Tensile tests

Following the ASTM D3039 standard [28], uniaxial tensile tests were performed using an Instron 5982 universal testing machine at room temperature conditions. Engineering strain was measured with extensometers. The testing rates for tensile was set to 1.5 mm/min. The UTS was determined to provide reference data for selecting tensile-tensile fatigue stress levels.

#### 2.2.2 Compression tests

Compression tests were conducted according to ASTM D3410 standard [29], with a compression rate set at 1.5 mm/min. The gauge length of the compression specimen was 25 mm. Vertical inclinometers were employed during specimen installation to ensure that, under compressive loads, the specimen experienced uniaxial stress in the vertical direction. The obtained Ultimate Compressive Strength (UCS) serves as a reference for pressure-to-pressure fatigue loading.

#### 2.2.3 Fatigue tests

**Fig. 2:**
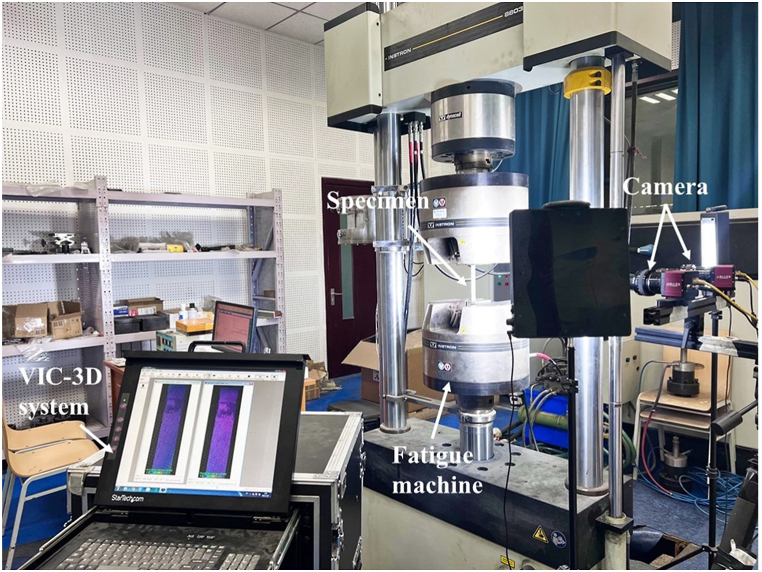
Setup of equipment during fatigue testing process.

As depicted in Fig. 2, fatigue tests were conducted using an Instron 8803 servohydraulic fatigue testing machine, employing a sinusoidal waveform with a constant 1 Hz frequency for loading. Based on mechanical properties obtained from uniaxial tensile and compression tests, fatigue test parameters were determined. For tensile-tensile (TT) fatigue tests, conducted following the ASTM D3479 standard [30], stress levels were set at 45 %, 55 %, and 65 % of the ultimate tensile strength (UTS). For compressive-compressive (CC) fatigue tests, stress levels were set at 55 %, 65 %, and 75 % of the ultimate compression strength (UCS). The minimum stress for TT fatigue tests was 0 MPa, and the maximum stress for CC fatigue was also 0 MPa. To ensure experimental accuracy, a vertical inclinometer was employed during specimen installation to measure and ensure perpendicularity. Additionally, multiple sets of repeated tests were conducted at each stress level, as detailed in Table 2. High-contrast, diffuse-reflection black-and-white speckle patterns on the cross-sectional surface of the specimens were achieved by spraying black paint onto a thin layer of white coating. Changes in the speckle pattern on the sample surface were monitored using the VIC-3D system, which utilizes a high-resolution CCD industrial camera with a spatial resolution of 96 dpi × 96 dpi, a frame rate of 3 fps, and an aperture ranging from f/5.6 to f/8.0. DIC techniques were employed to analyze the evolution of the surface strain field of the specimens under cyclic loading. Finally, Hitachi S–3400 N scanning electron microscopy was employed to examine specimen morphology after fatigue failure. The acceleration voltage was set to 15 kV, working distance ranged from 7 to 15 mm, and the magnification varied from 50 to 2000 times. To ensure the observation of non-conductive epoxy resin morphology, all samples underwent gold sputter coating.

**Table 2:** Fatigue test schemes and results.

No	Code	Fatigue loading methods	Stress ratio R	Maximum stress σ_max_ (MPa)		Fatigue life N_f_	Number of replicates
1	TT-45	Tensile-tensile	0	45%σ_t_/	113	1028000 (ongoing)	2
2						1001000 (ongoing)	
3	TT-55			55%σ_t_/	138	196412	3
4						213195	
5						231546	
6	TT-65			65%σ_t_/	163	27464	3
7						32421	
8						40549	
9	CC-55	Compressive-compressive	N/A	55%σ_c_/	79	1008600 (ongoing)	2
10						1005400 (ongoing)	
11	CC-65			65%σ_c_/	93	161214	3
12						181403	
13						219452	
14	CC-75			75%σ_c_/	107	86134	2
15						94955	

#### 2.2.4 Data Acquisition and processing methods

**Fig. 3:**
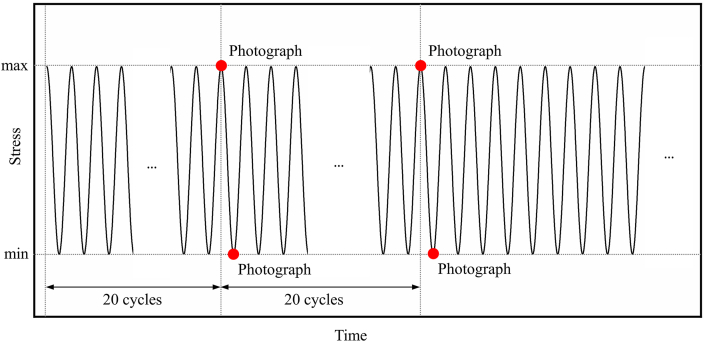
Schematic diagram of photograph taken at peak and valley points every 20 cycles.

Hysteresis loop data is acquired through the fatigue machine sensor. Balancing computational efficiency and the representativeness of sampled data involves selecting data from the first 100 cycles for every 1000 cycles. Each hysteresis loop dataset comprises 1000 stress-strain data points. The Fulcrum module is employed to link the fatigue machine and VIC-3D device, enabling the automatic collection of strain data at specific phases. As depicted in Fig. 3, speckle patterns on the specimen's surface are captured at the peaks and valleys (corresponding to maximum and minimum strains) after every 20 fatigue cycles (following the reference specimen's lifetime, with the photographing rate adjusted to 500 cycles^−1^ during the stable phase). This procedure aims to capture the initiation, propagation, and connection processes of fatigue cracks. Precise tracking of relative displacement between two points in the speckle patterns is achieved using a virtual extensometer. The maximum and minimum strains in the strain-cycle relationship presented are calculated based on the virtual extensometer.

## 3 Results and discussion

### 3.1 Tensile and compression curve analysis

**Fig. 4:**
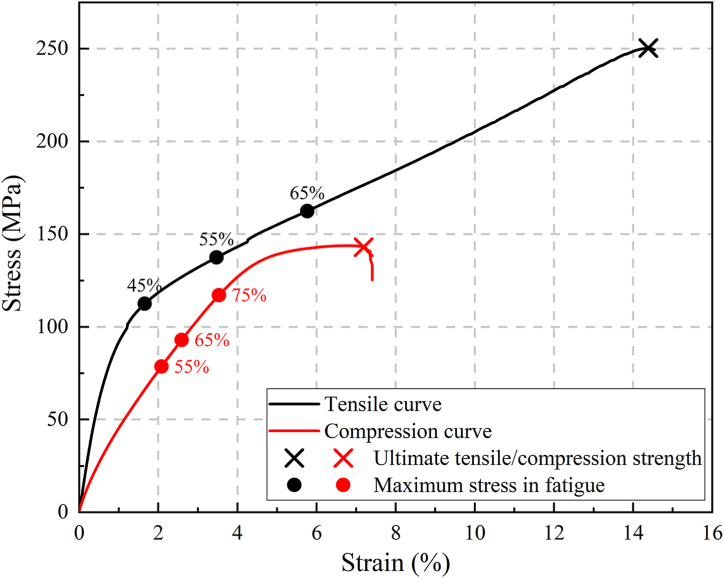
Stress-strain curves for uniaxial tensile and compression tests.

Fig. 4 illustrates the stress-strain curves for both tensile and compression tests. During the tensile testing, After reaching 45 % UTS, the stress uniformly increased with strain until failure. The UTS measured to be 250.4 MPa, with a fracture strain of 14.2 %. During the compression test, the stress-strain curve initially exhibited a linear relationship. Prior to fracture failure, the slope of the stress-strain curve rapidly decreased. The UCS was measured at 142.9 MPa, with a fracture strain of 7.2 %, as shown in Table 3. Comparatively, the tensile and compression curves exhibit significant differences, showing noticeable tensile-compression asymmetry.

**Table 3:** Mechanical properties of uniaxial tensile and compression.

UTS (MPa)	Young's modulus (GPa)	Tensile fracture strain (%)	UCS (MPa)	Compression modulus (GPa)	Compression fracture strain (%)	Number of replicates
250.37(5.31)	13.25(0.36)	14.5(0.03)	142.91(4.92)	6.16(0.28)	7.2(0.08)	5 resp.

### 3.2 Analysis of fatigue damage behavior

#### 3.2.1 Evolution of fatigue performance

**Fig. 5:**
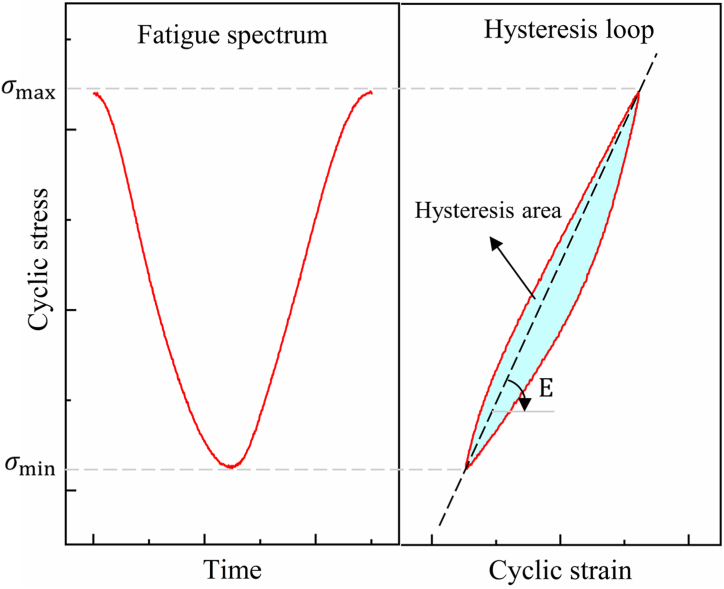
Cyclic loading and hysteresis loop example.

In Fig. 5, a single-cycle load history and the corresponding hysteresis loop are illustrated. The hysteresis loop represents closed stress-strain curves that deviate during loading and unloading cycles in fatigue tests. These loops form due to alterations in the composite material's microstructure. CFRP composites comprise fibers and a matrix, which inherently contain microscopic defects and cracks, leading to localized stress concentration during loading. As the material undergoes loading, it encounters internal resistance while adapting to the new strain state. During unloading, irreversible microstructural changes leave some strain unreleased, resulting in incomplete strain paths and the formation of hysteresis loops. By calculating the slope of the hysteresis loop, the residual modulus ratio (E/E_0_) can be obtained, and the area of the hysteresis loop represents the single-cycle dissipated energy (DE) density, as shown in Eq. (1).

(1) $$w_{d}=\int \sigma d\varepsilon =\int_{0}^{2\pi /\varpi \left(=1/f\right)}\sigma \left(t\right)\dot{\varepsilon }dt$$

where $w_{d}$ represents the single-cycle dissipated energy, σ is stress, ε is strain, ω is angular frequency, f is frequency, and t is time.

##### 3.2.1.1 For the tensile-tensile fatigue

**Fig. 6:**
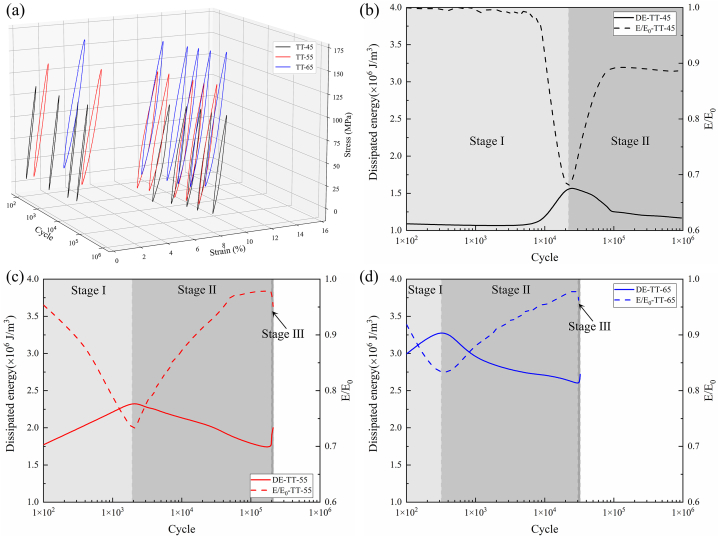
Evolution of tensile fatigue performance. (a) hysteresis loop results at different stress levels; (b), (c), and (d) evolution of energy dissipation and residual modulus ratio for tensile-tensile fatigue at 45 %, 55 %, and 65 % stress levels.

**Fig. 7:**
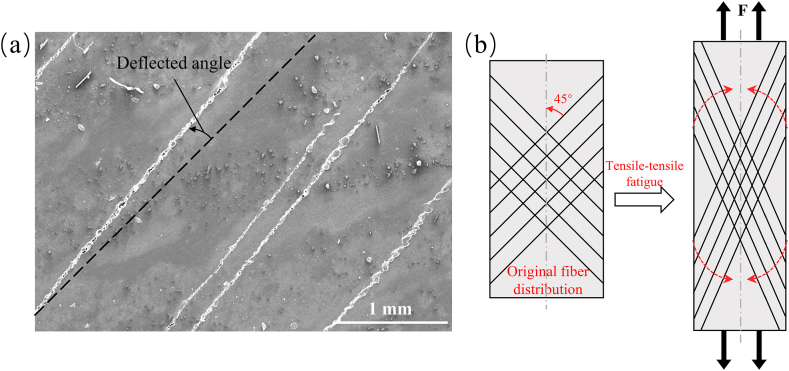
Fiber deflection convergence (a) phenomenon and (b)schematic in tensile-tensile fatigue.

As shown in Fig. 6(a), hysteresis loops under fatigue loading for tensile-tensile loading at different stress levels are depicted. The variation patterns of single-cycle dissipated energy density and residual modulus ratio are determined by calculating the hysteresis loop area and slope. Fig. 6(b)–(d) show the changes in dissipated energy density and residual modulus ratio with the number of cycles for tensile-tensile fatigue at stress levels of 45 %, 55 %, and 65 %. It is generally believed that carbon fiber composite materials experience stiffness degradation and an increase in single-cycle dissipated energy under cyclic loading [31,32]. However, this is not entirely applicable to off-axis CFRP composites due to the fiber deflection effect in off-axis ply layups. During the tensile-tensile fatigue process, the material undergoes three stages of performance evolution. The first stage is the damage initiation stage, during which initial performance degradation occurs. Microcracks initiate within the matrix and at the fiber/matrix interface, causing localized stiffness reduction and a rapid overall modulus decrease [33]. Consequently, there is a swift increase in dissipated energy per single cycle. The second stage is the steady resilience stage. Here, under the influence of cyclic loading, micro-damage gradually extends and connects, forming larger crack and damage regions [34]. Simultaneously, the fiber's deflection effect becomes active. As shown in Fig. 7(a) and (b), the dashed line signifies the 45° direction, under the fatigue tensile stress, the fibers gradually converge in the loading direction, enhancing the material's stiffness in that direction. This counteracts the modulus reduction caused by the damage area, resulting in a modulus increase during this stage and a decrease in single-cycle dissipated energy. The third stage is the final deterioration stage, which occurs just before failure. During this stage, cracks propagate rapidly, accompanied by a significant amount of fiber/matrix interface debonding and pull-out. These processes collectively contribute to severe structural damage, resulting in a decrease in modulus and an increase in dissipated energy. Ultimately, these effects culminate in final failure. At a stress level of 45 %, after going through the steady resilience stage and due to the relatively low stress level, the material did not enter the final deterioration stage, and no fracture failure occurs after undergoing 10^6^ cyclic loads. At a stress level of 55 %, the three stages accounted for 0.9 %, 90.6 %, and 8.5 % of the total fatigue life, respectively. When the stress level increased to 65 %, the three stages accounted for 0.9 %, 91.0 %, and 8.1 % of the total fatigue life, respectively. As shown in Table 4, when the stress level increased from 45 % to 65 %, the single-cycle average dissipated energy increased from 1.21 J/m³ to 2.69 J/m³. However, the average residual modulus ratio also increased from 0.88 to 0.95. This can also be attributed to the fiber deflection effect under tensile stress. As the fatigue stress level increased, the fiber deflection angle increased, thereby increasing the stiffness in the loading direction.

**Table 4:** Average dissipated energy density and average residual modulus ratio of tensile fatigue.

Code	Average dissipated energy density (J/m³)	Average residual modulus ratio
TT-45	1.21	0.88
TT-55	1.94	0.96
TT-65	2.69	0.95

##### 3.2.1.2 For the compressive-compressive fatigue

**Fig. 8:**
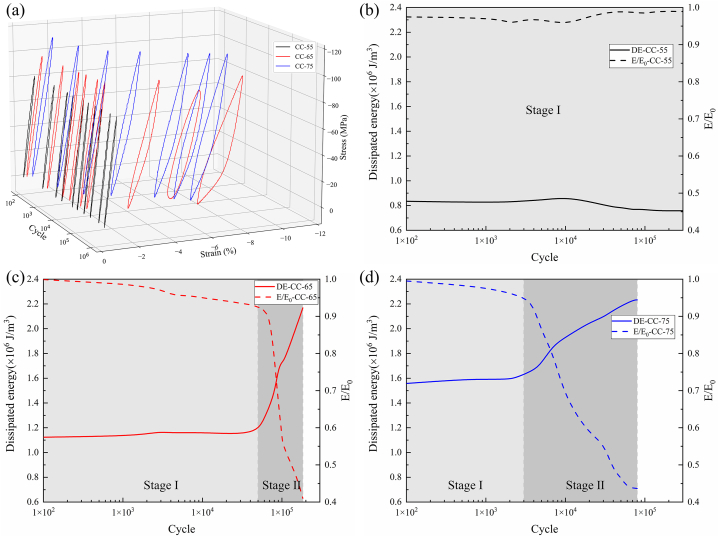
Evolution of compressive fatigue performance. (a) hysteresis loop results at different stress levels; (b), (c), and (d) evolution of energy dissipation and residual modulus ratio for compressive-compressive fatigue at 55 %, 65 %, and 75 % stress levels.

**Fig. 9:**
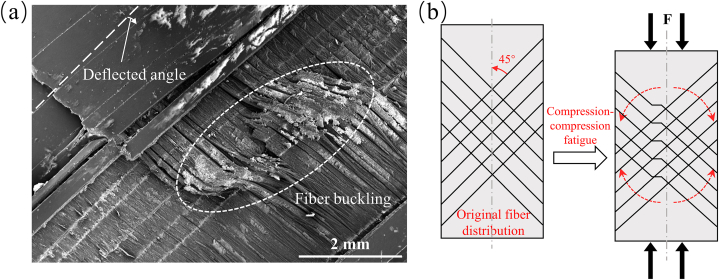
Fiber deflection buckling (a) phenomenon and (b) schematic in compressive-compressive fatigue.

As shown in Fig. 8(a), hysteresis loops under fatigue loading for compressive-compressive loading at different stress levels are depicted. The compressive-compressive fatigue performance evolution can be categorized into two stages based on the variations of dissipated energy and residual modulus ratio with the number of cycles. The first stage is characterized by the initiation of microcracks in the matrix under compressive loading, accompanied by a degradation in stiffness and an increase in dissipation energy density. The second stage is the fiber buckling stage, during which the fibers begin to deflect and buckle, leading to non-uniform stress distribution in the fiber direction. Under the influence of cyclic loading, more fiber misalignment occurs, ultimately resulting in failure. As shown in Fig. 8(b), at a stress level of 55 %, the material exhibits minimal variations in modulus and dissipated energy, with no occurrence of fracture failure after 10^6^ cycles. However, when the stress level reaches 65 % and 75 %, localized stress concentrations caused by matrix defects, cracks, and interlayer shear promote the accumulation of damage. Consequently, as shown in Fig. 8(c) and (d), the modulus continues to decrease, while the dissipated energy keeps increasing, ultimately leading to failure. As shown in Fig. 9(a) and (b), localized fiber buckling occurs at failure as the damage progresses, which is the main factor leading to compression fatigue failure [35]. As shown in Table 5, with increasing compressive stress levels, the single-cycle dissipated energy increases from 0.77 J/m³ to 2.1 J/m³, while the residual modulus ratio decreases from 0.99 to 0.55. During compression, the fiber deflection effect no longer promotes the recovery of material performance but instead accelerates stiffness degradation.

**Table 5:** Average dissipated energy density and average residual modulus ratio of compressive fatigue.

Code	Average dissipated energy density (J/m³)	Average residual modulus ratio
CC-55	0.77	0.99
CC-65	1.59	0.70
CC-75	2.10	0.55

#### 3.2.2 Crack propagation and failure mechanisms

##### 3.2.2.1 For the tensile-tensile fatigue

**Fig. 10:**
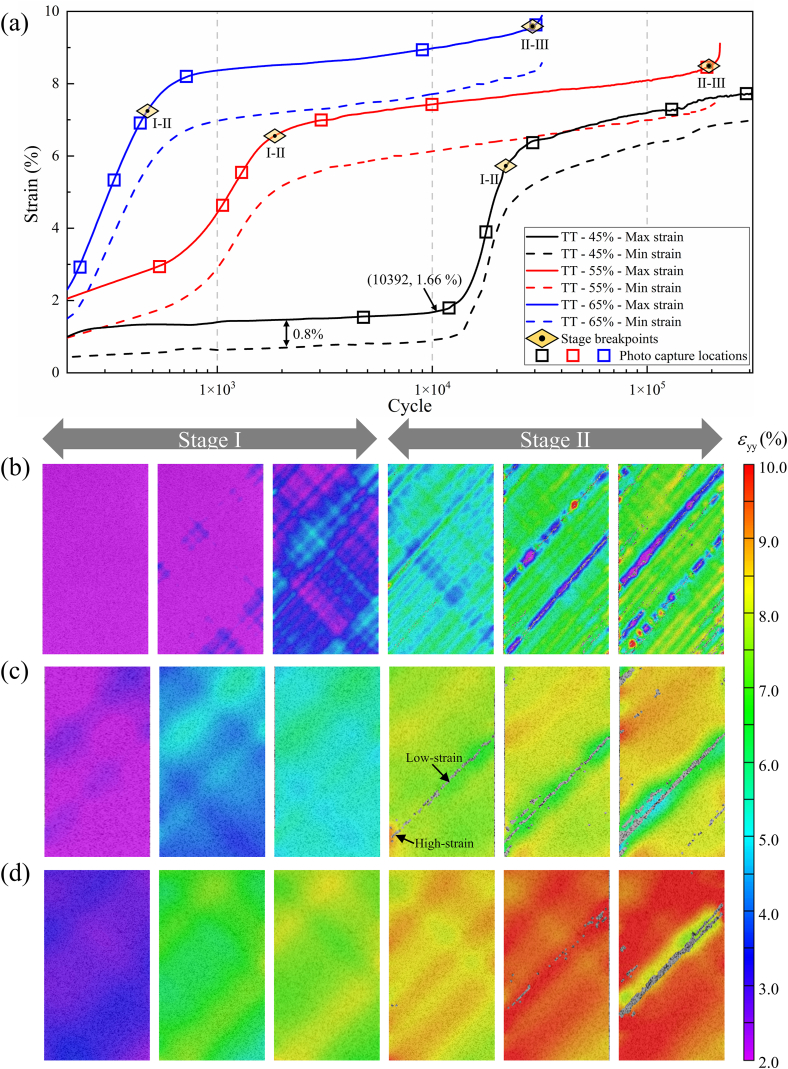
Strain-cycles curves and DIC images of tensile-tensile fatigue process. (a) strain-cycles curves; (b), (c), and (d) full-field axial strain distributions for stress levels of 45 %, 55 %, and 65 %, respectively.

**Fig. 11:**
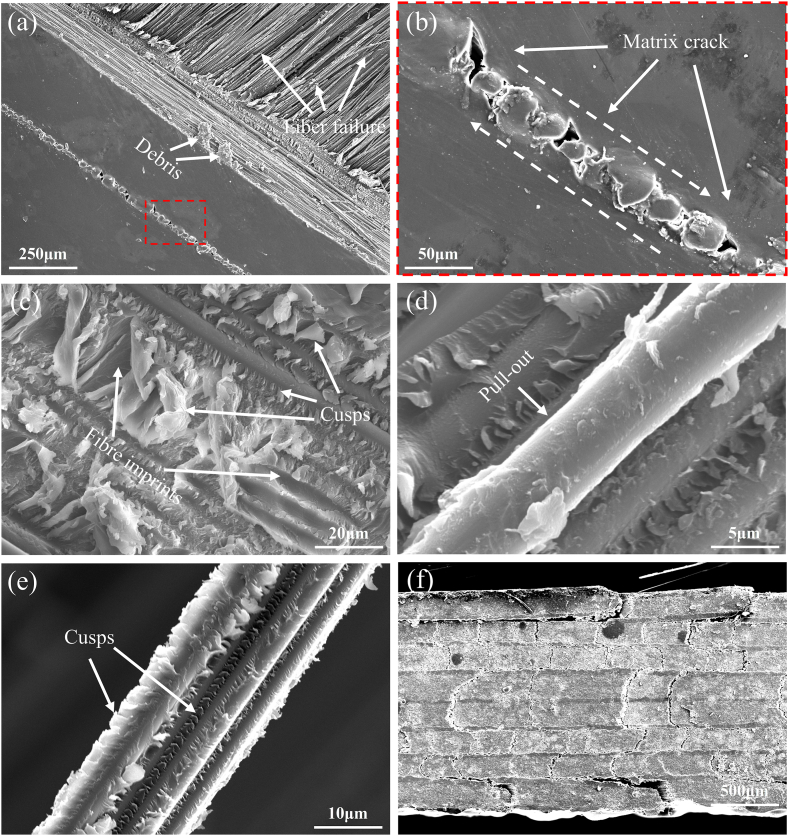
Failure morphology of tensile-tensile fatigue. (a) and (b) shear damage; (c), (d) and (e) fiber debonding and pull-out marks at the fracture surface; (f) delamination morphology away from the fracture surface.

In Fig. 10(a), during tensile-tensile fatigue testing at a stress level of 45 %, the strain curve can be divided into two stages: the damage initiation stage and the steady resilience stage. In the damage initiation stage, the strain rapidly stabilizes, with a strain difference of approximately 0.8 %. It can be observed that the specimen has undergone plastic deformation by examining the minimum strain. When the loading reaches 10392 cycles, the material gradually becomes unstable, with a strain of approximately 1.66 %. Referring to the tensile stress-strain curve in Fig. 4, the stress corresponding to the 1.66 % strain is approximately 45 % UTS. Subsequently, the strain increases rapidly, reaching a plateau at around 20000 cycles, entering the steady resilience stage. In this stage, the material's stiffness recovers, and the strain amplitude decreases, and no fracture failure occurs after undergoing 10^6^ cyclic loads. At stress levels of 55 % and 65 %, the specimens go through three stages. Initially, there is a rapid increase in strain during the damage initiation stage, followed by the steady resilience stage with a slower strain growth rate. With continued loading, they eventually enter the final deterioration stage, leading to fracture. In Fig. 10(b), (c), and (d), DIC images for tensile-tensile fatigue at stress levels of 45 %, 55 %, and 65 % are provided. These images capture the y-direction strain fields during rapid strain variations, illustrating the evolution of surface deformation fields. It is evident that as the stress level increases, the specimen's surface transitions from individual or isolated fiber bundles to a larger number of fiber bundles that exhibit improved continuity and strong adhesion to the matrix. With an increasing number of cycles, the specimen subjected to 55 % stress level exhibits variations in strain along the fiber direction. This is evident through the emergence of high-strain regions at the edges and low-strain regions at the center, leading to delamination and cracks between these regions. As delamination and cracks continue to propagate, new stress concentration areas develop on both sides, effectively dispersing stress from the previously damaged region and reducing the rate of crack propagation. For 65 % stress level, as cracks propagate and extend, the specimen eventually fractures directly without further stress concentration area transfer. Combining the fatigue strain curves and DIC images reveals that during the damage initiation stage of tensile-tensile fatigue, the specimen's surface gradually transitions from uniform loading to localized stress concentration. This results in a rapid overall strain increase until noticeable stress concentration regions appear. Subsequently, the specimen enters the steady resilience stage. During this stage, the strain variation in the area of interest (AOI), defined as the central region measuring 25 mm × 37.5 mm on the specimen, becomes less pronounced due to the fiber deflection effect. The cracks in the stress concentration region gradually extend and open, resulting in a slowdown in overall strain changes. Finally, when the specimen enters the final deterioration stage, delamination, fiber/matrix debonding, and the formation of penetrating shear cracks along the fiber direction, as shown in Fig. 11 (a) - (f), lead to the pull-out of a significant number of fibers, resulting in fatigue failure.

**Fig. 12:**
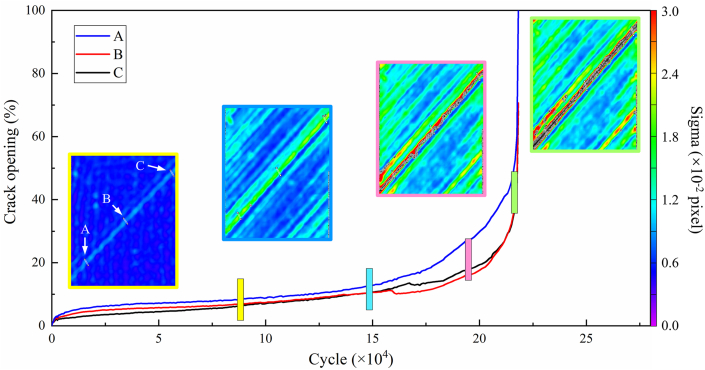
Crack propagation path in tensile-tensile fatigue during the cyclic loading.

Sigma represents the confidence interval of pixel matching on the specimen's surface, facilitates the expedited identification of defects and damage. The use of Sigma as a variable in the DIC images provides a quantitative characterization of the crack propagation process. To elucidate the mechanisms from the appearance of strain concentration regions along the fiber direction to the formation and connection of cracks, virtual extensometers were established perpendicular to the cracks at three positions: left (A), middle (B), and right (C) along the crack surface. The relationship between crack opening and the number of cycles was established at these three positions, as depicted in Fig. 12, it was observed that all three positions experienced crack opening. However, positions B and A showed subsequent decreases in opening size after 159165 cycles. This phenomenon occurred because the defect size at position C was consistently larger than at the other locations, leading to the formation of a crack centered around the C defect, gradually connecting to the B defect during the cyclic process. Before the B defect connected to the C crack, it underwent a brief stress release stage, causing a temporary contraction of the crack. Once the B defect connected to the C crack, the crack size at position B followed the changes in the C crack. Similarly, at 167168 cycles, the decrease in opening size at position A was influenced by the extension and connection process of the C crack. Afterward, the opening size of the crack at position A followed the trend of the C crack. Therefore, for off-axis laminated CFRP, cracks propagate by forming multiple concurrent defects, with larger edge defects dominating and connecting smaller defects, eventually forming penetrating cracks along the fiber direction.

##### 3.2.2.2 For the compressive-compressive fatigue

**Fig. 13:**
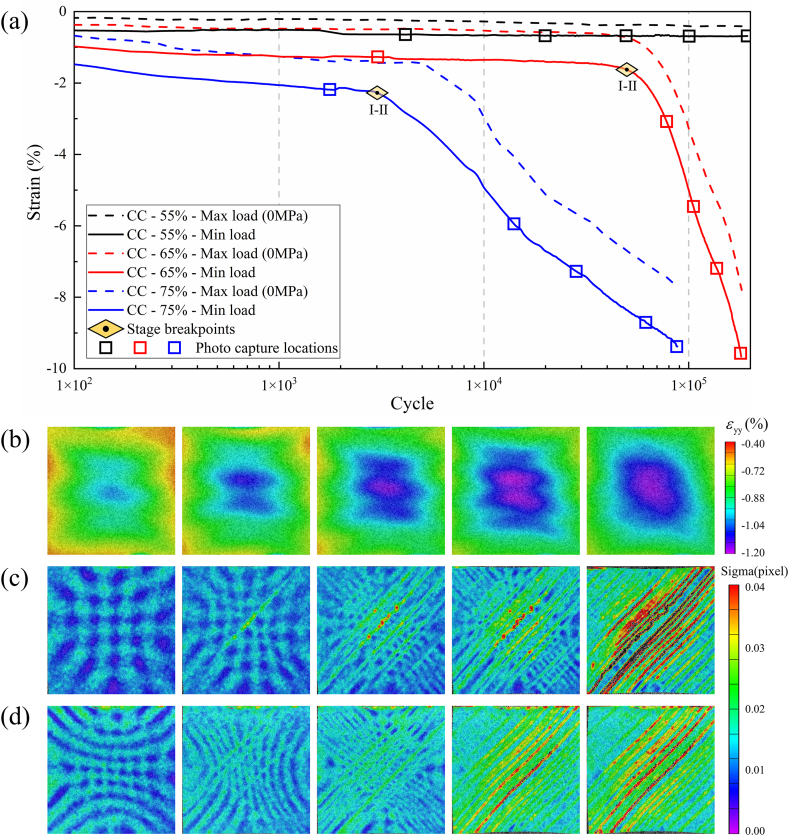
Strain-cycles curves and DIC images of compressive-compressive fatigue process. (a) strain-cycles curves; (b) full-field axial strain distributions for stress levels of 55 %; (c) and (d) the sigma values for stress levels of 75 % and 65 %.

**Fig. 14:**
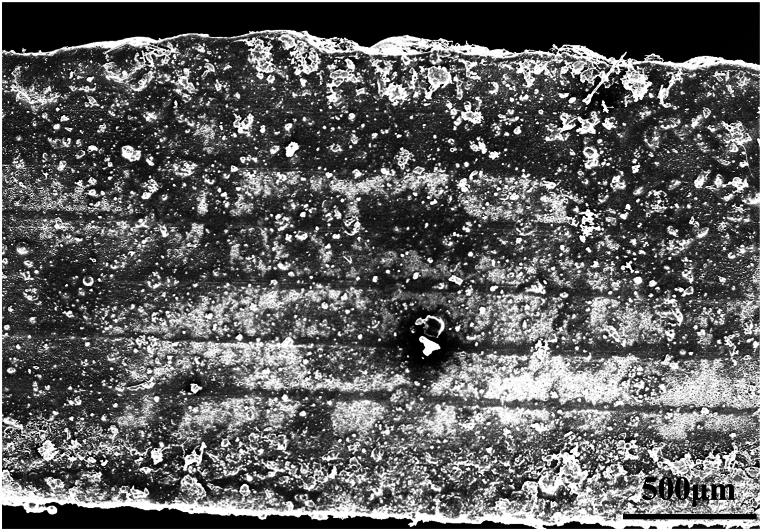
Side view of the specimen at 55 % stress level with minimal observable damage.

**Fig. 15:**
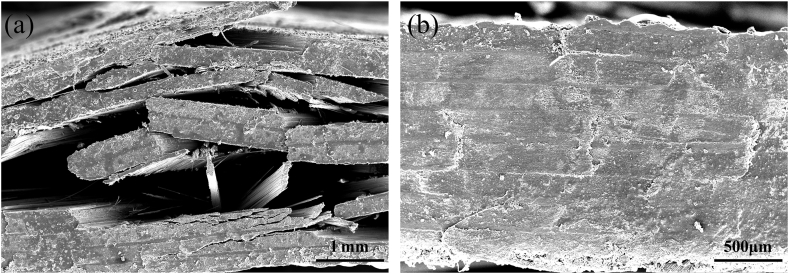
Failure morphology of compressive-compressive fatigue. (a) compressive failure at the fracture surface, (b) morphology away from the fractured surface.

In the case of compressive-compressive fatigue, as depicted in Fig. 13(a), when the stress level is set at 55 %, the specimen remains stable even after 10^6^ cycles of loading. The stress-strain curve remains stable, and there are no apparent signs of damage, as shown in Fig. 14. However, as the stress level is increased to 65 %, the specimen experiences a rapid increase in fatigue strain after 33965 cycles (18.7 % of N_f_) and ultimately fractures. Similarly, at a stress level of 75 %, the specimen starts to experience a rapid increase in fatigue strain after 2865 cycles (3.3 % of N_f_) and fractures after 87955 cycles. Notably, the fatigue fracture strains at 65 % and 75 % stress levels, signifying the maximum strains at the point of fracture, are comparable, measuring −7.75 % and −7.79 %, respectively. Additionally, the specimens fail directly after experiencing rapid strain changes. This is because in compressive-compressive fatigue, fiber buckling is the primary reason for strain changes and specimen failure [[36], [37], [38]]. Therefore, this fracture strain can be considered one of the indicators of failure in compressive-compressive fatigue. Fig. 13(b) illustrates the y-direction surface strain field at a 55 % stress level. It can be observed that during loading, the strain maintains a good central symmetric appearance, with the maximum strain at the center. This indicates good fatigue clamping, ensuring that the material experiences uniaxial stress in the vertical direction. Fig. 13(c) and (d) show the surface sigma variation fields at stress levels of 65 % and 75 %. In the initial stages of loading, they exhibit centrally symmetric spotted patterns. As loading progresses, tangential high-sigma regions appear, indicating significant shear effects in the central region, leading to crack defects along the fiber direction. With the progression of the fatigue process, shear damage gradually spreads from the center to the edges, forming a pronounced "S"-shaped distribution, signifying significant fiber buckling in the compressive-compressive process. Combining the strain curves and DIC images, it can be seen that the gradual strain change corresponds to the stress concentration process in the specimen, indicating the damage initiation stage. The accelerated strain change corresponds to the damage expansion process, indicating the fiber buckling stage. Furthermore, as shown in Fig. 15(a) and (b), it can be observed that there is minimal delamination damage in the compressive-compressive fatigue process, with most of the shear damage occurring within the layers. As shear damage accumulates, the outer-layer fibers first undergo buckling and fracture, followed by further buckling of the inner fibers due to instability, ultimately leading to material failure through compression.

## 4 Conclusions

(1) The degradation of fatigue performance is influenced by a combination of factors, including matrix defects, microcrack propagation, shear damage accumulation, and a decrease in fiber/matrix interface strength. It is noteworthy that the deflection and buckling of off-axis fibers play a pivotal role in causing fatigue failure in compressive-compressive scenarios. However, in the tensile-tensile fatigue process, the fiber deflection effect exhibits a strengthening role, manifested as the recovery of stiffness and a reduction in dissipated energy.
(2) Tensile-tensile fatigue can be divided into three stages: damage initiation, steady resilience, and final deterioration. In the damage initiation stage, the presence of internal defects and voids triggers the formation of localized microcracks, resulting in a rapid decrease in stiffness. The steady resilience stage accounts for over 90 % of the fatigue life, where the stiffness degradation caused by cumulative damage is balanced by the stiffness recovery induced by fiber deflection. Therefore, stiffness strengthens and exhibits an upward trend during this stage. In the final deterioration stage, large edge cracks dominate and connect with internal microcracks, forming extensive shear cracks spanning the entire fiber direction. This leads to the debonding of the fiber/matrix interface, ultimately resulting in failure.
(3) Compressive-compressive fatigue can be divided into two stages: the damage initiation stage and the fiber buckling stage. The damage initiation stage is similar to tensile-tensile fatigue. However, in the case of compressive-compressive fatigue, during the fiber buckling stage, the crack propagation path extends from the center towards the edges. Initially, shear cracks form in the central position within the layer and then propagate towards the edges along the fiber direction, resulting in the characteristic 'S'-shaped fiber buckling and eventual failure. Additionally, a compressive strain threshold of 7.8 % was determined for the off-axis CFRP composites. This threshold can serve as a failure criterion for compression testing or compressive-compressive fatigue testing, providing a valuable reference for material performance prediction and engineering applications.

## CRediT authorship contribution statement

**Zhendong Zhong:** Writing – original draft, Methodology. **Fusheng Wang:** Methodology, Investigation, Data curation. **Fanqi Kong:** Writing – original draft, Investigation. **Yajun Chen:** Writing – review & editing, Supervision, Conceptualization.

## Declaration of competing interest

The authors declare that they have no known competing financial interests or personal relationships that could have appeared to influence the work reported in this paper.
